# Supplementary material for: Knowledge, attitude, and hesitancy towards COVID-19 vaccine among university students of Bangladesh
Source: PLoS One. 2022 Jun 27;17(6):e0270684. doi: 10.1371/journal.pone.0270684 (PMC9236250; doi:10.1371/journal.pone.0270684)
Supplement: S1 Questionnaire — (DOCX) [file pone.0270684.s001.docx]

Perception towards Covid-19 Vaccination among University Students of Bangladesh

This survey will take 05~08 minutes. Your Contribution will be highly appreciated! In this survey you will be asked some questions to evaluate your perception towards COVID-19 vaccination. Only university students of Bangladesh can participate in this survey. Please read the questions very well and provide your answers accordingly. This research has maintained all the ethical issues. Please note that all the information you provide us is totally CONFIDENTIAL and would be used only by the research team for the purposes of research. If you Agree to participate in this survey, please continue the next section. You are free to leave this survey any time. For further details please contact the research team.

**Socio-demographic and Academic Information**

1. Gender

- Male
- Female

1. Marital Status

- Married
- Unmarried

1. Are you living with Family?

- Yes
- No

1. Where do you live in?

- Dhaka
- Outside Dhaka

1. Did you have the COVID-19?

- Yes
- No

1. Which media you use most for COVID-19 vaccine and precautionary measures

related information?

- University
- Internet
- Electronic media (TV, Radio)
- Social Media
- Print Media
- People (Community, Family Members)

1. University

- Public
- Private

1. Year

- 1st
- 2nd
- 3rd
- 4th and Masters

1. Degree Major

- Science and Engineering
- Arts and Social Science
- Business and Economics
- Security and Strategic
- Medical Studies

Knowledge

1. COVID-19 vaccines are effective at keeping you from getting COVID-19

- True
- False
- I don't know

1. You need to maintain the regulations of preventing COVID-19 after being vaccination

- True
- False
- I don't know

1. Getting a COVID-19 vaccine will also help keep you from getting seriously ill even if you get COVID-19

- True
- False
- I don't know

1. People who have been fully vaccinated can start to do some things that they had stopped doing because of the pandemic

- True
- False
- I don't know

1. There is a website where you can apply for vaccination in Bangladesh

- True
- False
- I don't know

1. Like all other vaccines, this vaccine has the potential for some side

effects

- True
- False
- I don't know

1. If there are side effects due to COVID-19 vaccination, they normally go away in a few days

- True
- False
- I don't know

1. The COVID-19 vaccine can create infertility

- True
- False
- I don't know

1. The COVID-19 vaccine can create long term physical problems

- True
- False
- I don't know

Attitude

1. I am concerned about the COVID-19 pandemic

- Agree
- Neutral
- Disagree

1. I think that the COVID-19 vaccine is safe and effective

- Agree
- Neutral
- Disagree

1. If I am eligible for vaccine, I need to take it as soon as possible

- Agree
- Neutral
- Disagree

1. My family members and neighbors should take the vaccine, and I should aware and motivate them to take vaccine.

- Agree
- Neutral
- Disagree

1. I think vaccination will help us to stop spreading COVID-19

- Agree
- Neutral
- Disagree

Hesitation

1. Are you hesitated to take COVID-19 vaccine?

- Yes
- No

Reason behind hesitation to take COVID-19 Vaccine

1. I am hesitated to take COVID-19 vaccine because I don't think I am eligible for it

- True
- False

1. I am hesitated to take COVID-19 vaccine because I don't know enough about it

- True
- False

1. I am hesitated to take COVID-19 vaccine because I am afraid of the side effects

- True
- False

1. I am hesitated to take COVID-19 vaccine because I may have to pay money for it

- True
- False

1. I am hesitated to take COVID-19 vaccine because it is against my religious believe

- True
- False

1. I am hesitated to take COVID-19 vaccine because of other reasons

- True
- False
