# Supplementary material for: Knowledge, attitude, and hesitancy towards COVID-19 vaccine among university students of Bangladesh
Source: PLoS One. 2022 Jun 27;17(6):e0270684. doi: 10.1371/journal.pone.0270684 (PMC9236250; doi:10.1371/journal.pone.0270684)
Supplement: Attachment — Submitted filename: Rebuttal letter.docx [file pone.0270684.s002.docx]

**Response to Reviewers**

1. The authors does not follows PLOS standard guidelines in preparing the manuscript. Please visit and read website and prepare the manuscript according to journal standard guidelines.

**Response**: Thank you for the important point. We are extremely sorry that we missed some points mentioned in the standard guidelines. We have revised our manuscript again following the journal standard guidelines (please check track changes and clean version). We have also changed some other parts to fit the manuscript (converted Fig 1 to Table 2 and mentioning Ethical Clearance reference number). We hope that the current version would satisfy you.
