# Supplementary material for: Knowledge, attitude, and hesitancy towards COVID-19 vaccine among university students of Bangladesh
Source: PLoS One. 2022 Jun 27;17(6):e0270684. doi: 10.1371/journal.pone.0270684 (PMC9236250; doi:10.1371/journal.pone.0270684)
Supplement: Attachment — Submitted filename: PONE-D-21-17512. report.docx [file pone.0270684.s003.docx]

This manuscript is noteworthy in the current era of COVID-19 pandemic because the issue of vaccine hesitancy is extremely important as WHO considers vaccine hesitancy as a significant threat to global health. The COVID-19 vaccine is one of the top priorities in the battle against the pandemic, but its effectiveness is dependent on individual decisions since vaccines are voluntary. Apart from scarcity in dose availability and inequity in vaccine delivery, vaccine hesitancy is one of the most serious challenges in this regard. A main finding of the study, that there is still a considerable vaccine hesitancy among university students despite their goog knowledge and positive attitude, which is consistent with studies in other countries and across broad demographic groups.

However, some issues needed to be addressed first to improve the quality of the manuscript: though, the following are general outlines of the required modifications:

Title: It is better to replace "hesitation" with "hesitancy" also along the whole manuscript.

- Language editing is needed for multiple issues
- **Introduction:**
- It would be good to provide a background on COVID-specific reasons for vaccine hesitancy, along with the general ones.
- Some background on vaccination plans in the Bangladesh should be displayed such as What vaccines are being considered, is the vaccination program already planned, number of currently vaccinated person. What is the perception of vaccines in Bangladesh, e.g., in the news? These are all crucial factors playing a role in vaccine hesitancy and affecting its dynamics.
- The rationale, study hypothesis and the study questions should be clearly stated

Methodology section: issues in the study design and settings.

**Methods:**

- " The current survey was performed among university students"
- Each group should be defined; by mentioning the study plan of each faculty and whether their curriculum contain COVI-19 vaccination topics or not?
- Also sample size calculation and sampling technique (details of methods of selection of participants) are not mentioned in the manuscript.
- **Results:**
- What is the age of the participants?

Knowledge leveling is either Good or poor or adequate & inadequate NOT positive or negative as the attitude

What is the level of COVID-19 vaccination hesitancy? Where this percentage in the results?? It needs a separate table as knowledge and attitude as it is one if the study objectives

- **Discussion:**
- Generally, the discussion is somewhat redundant without adequate structure and should be focused to compare the results with similar studies and to explain and give the implications of the study results.
- Please make the discussion more concise, without discussing too many details but the most important results and their general meaning.
- Having some bullet points on how to address the observed hesitancy would be beneficial for the discussion and the entire manuscript.
- **References:**
- Needs revision as sometimes they are not consistent in their style. Some of them are incomplete

**Decision**

- Consider after Major Changes
